# Digital Play Addiction Tendency and Aggressive Behaviors Among Turkish Preschoolers: Evidence from Parent Reports

**DOI:** 10.3390/ejihpe15110233

**Published:** 2025-11-14

**Authors:** Selahattin Semiz, Yüksel Büşra Yüksel Aykanat, Büşra Somuncu Çoksağır, Amira Mohammed Ali, Carlos Laranjeira, Murat Yıldırım

**Affiliations:** 1 Department of Early Childhood Education, Ağrı İbrahim Çeçen University, Ağrı 04100, Turkey; 2 Department of Early Childhood Education, Erciyes University, Kayseri 38280, Turkey; 3 Department of Early Childhood Education, Anadolu University, Eskişehir 26470, Turkey; 4 Department of Psychiatric Nursing and Mental Health, Faculty of Nursing, Alexandria University, Smouha, Alexandria 21527, Egypt; 5 School of Health Sciences, Campus 2, Polytechnic University of Leiria, Morro do Lena, Alto do Vieiro, Apartado 4137, 2411-901 Leiria, Portugal; 6 Centre for Innovative Care and Health Technology (ciTechCare), Polytechnic University of Leiria, Campus 5, Rua das Olhalvas, 2414-016 Leiria, Portugal; 7 Comprehensive Health Research Centre (CHRC), University of Évora, 7000-801 Évora, Portugal; 8 Department of Psychology, Faculty of Science and Letters, Ağrı İbrahim Çeçen Üniversitesi, Ağrı 04100, Turkey; 9 Psychology Research Center, Khazar University, Baku AZ1000, Azerbaijan

**Keywords:** digital play addiction tendency, aggression, early childhood, digital game

## Abstract

The escalating exposure of young children to digital gaming necessitates a critical examination of its behavioral impacts. However, evidence regarding its influence on aggressive behavior remains limited. This study investigated the relationship between digital play addiction tendency and our dimensions of aggression: physical aggression, relational aggression, self-directed aggression, and aggression against objects. This study employed a cross-sectional design, gathering data through parent assessments. The sample consisted of 744 children aged 4 to 6 years. The average age of the participants was 33.5, with 82% of the sample being female. The participants came from a lower (27%), middle (37%), and high (36%) socioeconomic background. The data were analyzed using a Structural Equation Modeling (SEM) approach to test the hypothesized relationships. The main findings from the SEM analysis indicated that a higher digital play addiction tendency was a significant positive predictor of all four dimensions of aggression. These results highlight the potential adverse effects of digital play addiction tendency on the development of maladaptive behaviors in early childhood. This study underscores the urgent need to develop strategies that foster healthier digital media consumption and mitigate the adverse effects of digital gaming on children’s developmental outcomes.

## 1. Introduction

Play is foundational to early childhood development, serving as the primary medium through which children explore the world, cultivate social skills, and engage in imaginative learning (Hirsh-Pasek et al., 2009). While traditional games have been the cornerstone of childhood entertainment and education for centuries, the contemporary environment has undergone a dramatic shift. Fueled by rapid technological advancements, young children are now immersed in a diverse digital world from an early age, with information and communication technologies readily accessible within the home. This pervasive exposure has fundamentally transformed the landscape of childhood play, establishing technology as an increasingly integral component of their daily lives (e.g., Organisation for Economic Co-Operation and Development (OECD), 2019; Marsh et al., 2020; Tena et al., 2019).

The ubiquity of digital devices, particularly smartphones and tablets, has increased the accessibility and appeal of digital games, often leading children to prefer them over traditional forms of play (Işıkoğlu et al., 2023). Digital play can take various forms, encompassing both solitary and collaborative engagement. While some digital games foster creativity, teamwork, and strategic thinking, others may contribute to isolation, dependency, and reduced socio-emotional well-being (UNICEF, 2025). For instance, one study highlighted that young children’s proficiency with digital devices can surpass fundamental motor skills, such as riding a bike or swimming (Palaiologou, 2016). However, the design of digital games differs significantly from traditional play, which can shape their impact and occasionally lead to unintended consequences. A primary concern is the potential for developing a digital play addiction tendency. In early childhood, this tendency is not solely defined by the duration of use, but by a cluster of observable behaviors. These include preoccupation with the game when not playing, displaying irritability or distress when access is denied, loss of interest in other age-appropriate activities, and using gameplay to escape negative moods (Gansner, 2019). Factors such as early exposure to gaming or device ownership at a young age can exacerbate this risk (Weinstein, 2010).

These behavioral risks underscore the rationale behind clinical guidelines for screen time in early childhood. For instance, the American Academy of Pediatrics [AAP] Council of Communications and Media (2016) recommends that screen time for children aged two to six should not exceed one hour per day. Despite this guidance, the immersive qualities of digital games—from their vibrant graphics to captivating characters—can foster the very addictive tendencies that lead to prolonged engagement, pushing children well beyond these recommended limits. Consequently, this extended exposure has become a focal point of concern regarding its potential psycho-social impacts. While research has linked frequent gameplay to disruptions in academic skills and heightened emotional reactions (Taylan et al., 2017), a critical area of investigation is its influence on aggression. This study examines explicitly four dimensions of aggression potentially shaped by these gaming behaviors: physical aggression (PA), relational aggression (RA), self-directed aggression (SA), and aggression against objects (AO).

To provide a theoretical lens for this examination, the current study is grounded in Social Cognitive Theory (SCT) (Bandura, 1986, 2001), which provides a valuable framework for understanding how children’s behaviors are shaped through the dynamic interaction of personal, behavioral, and environmental factors. According to SCT, children acquire and regulate behaviors by observing others, imitating modeled actions, and responding to reinforcement and feedback. In the context of digital games, repeated exposure to in-game actions, rewards, and challenges may influence patterns of play, emotional responses, and tendencies toward overuse. For example, children may model aggressive behaviors observed in games or develop habits reinforced by in-game success, which could contribute to increased engagement and potential addictive patterns (Gentile et al., 2011). Thus, SCT provides a theoretical lens for interpreting how digital game experiences may be linked to behavioral outcomes, such as aggression and digital play addiction, in children.

To understand the relationship between children’s aggression levels and digital game addiction tendency in early childhood, we draw on social cognitive theory, which suggests that individuals learn by observing others and that this learning occurs through reinforcement. When applied to the context of digital play addiction tendency and aggression in children, this theory suggests that exposure to aggressive behavior in digital games can lead to the internalisation of aggressive scripts and desensitization to violence, thereby increasing the likelihood of aggressive behavior in children (Nagar et al., 2025). Moreover, the theory also emphasizes the role of cognitive processes, such as attention, retention, and reproduction of observed behaviors, in shaping individuals’ responses to media influences (Granic et al., 2020; Prescott et al., 2018).

In the context of this theory, several rationales underlie our consideration of variables. The first of these is modeling. Children learn by imitating what they see around them (family, friends, media characters, etc.). Digital games featuring aggressive content have been shown to prompt children to emulate such behaviors. The second factor is reinforcement. Children learn based on the consequences of their behaviors. Behaviors that are rewarded or deemed pleasant in digital games can lead to their repetition in children similarly, if a child desires to play a game but is consistently directed towards digital media tools by their parents. In that case, the child may exhibit aggressive behaviors when unable to do so. Lastly, social interaction plays a critical role in shaping an individual’s behavior. Prolonged engagement with digital gaming may hinder children’s social skill development, potentially increasing tendencies towards aggression.

The adoption of this theory is driven by our quest to gain a nuanced understanding of how children’s digital game addiction correlates with their aggression levels. Social cognitive theory underscores the pivotal roles of observational learning, cognitive processes, and the impact of digital media in shaping children’s behaviors. By applying this framework, we aim to elucidate the intricate dynamics through which digital play addiction tendency may influence heightened aggression in children.

The early years of a child’s life form the basis of their lifelong development, and therefore, the experiences they acquire during this period become crucial. Among these experiences are digital technologies, which hold a crucial place in children’s daily lives today, and digital games, which serve as an extension of this. While digital games can offer positive opportunities such as rich, fun, and interactive experiences by supporting children’s learning, cognitive development (Boot et al., 2008), social interaction, and physical activities (Mhurchú et al., 2008), they can also offer experiences that negatively affect their academic achievement, development, and behavior. The habits children form through these experiences have the potential to influence their behavior. A recent example of this is that during the lockdown period due to the COVID-19 pandemic, children spent more time with technological devices because they were spending more time indoors (Koran et al., 2022; Limone & Toto, 2021). In the Turkish context, which we address in our study, statistical data also reflect a significant increase in digital participation among young children. The Turkish Statistical Institute reports (Turkish Statistical Institute, 2013, 2021) indicate that internet usage among 6–15-year-olds increased from 50.8% to 82.7% during the period encompassing the closure. Young children in this age group reportedly spend around 3 h daily on social media, with 96.2% of boys and 91.8% of girls engaging in digital gameplay. The increased use of digital devices during this period is associated with a higher tendency among children to play digital games, which in turn may contribute to patterns of overuse or digital game addiction.

Debates persist regarding the implications of young children’s increased technological engagement, capturing both curiosity and concern. The discourse surrounding the advantages and potential risks of technology in children’s daily lives has been a central theme in research and informal conversations for over a decade. Touchscreen devices have emerged as significant tools for engaging and entertaining children (Johnston, 2021). Türkiye ranks among the top countries where digital games are widely played (Turgut & Yaşar, 2019). While increased interaction with digital games has increased children’s creativity and skills, such as seeking ways to connect socially with others and adapting in a time of limited opportunities, such as the pandemic (Cowan et al., 2021), children have experienced problems such as aggression, problem behavior, and communication breakdowns (Rahayu et al., 2021). At this point, it has become imperative to determine the effect of digital play on children’s behavioral development. Early detection of digital play addiction tendency will help minimise such adverse effects in the future. Studies examining the effects of digital games at older ages are more intense (Ekinci et al., 2016; Kesici, 2020), while studies on digital games in early childhood focus on examining the relationship between screen usage time (Žulec et al., 2023) or digital play and parental factors (Emiroğlu İlvan & Ceylan, 2023; Şenol et al., 2023).

Our study, however, examines the relationship between children’s digital play addiction tendencies and various aggressive behaviors, focusing on a relatively younger age group compared to many existing studies in the field. Unlike previous studies, which often focused on exploring the association between a single type of aggression and digital play addiction tendency (Caner & Evgin, 2021; Jeong et al., 2017; Rahayu et al., 2021; Şenol et al., 2023), our research focuses on examining sub-dimensions of aggression (PA, RA, SA, AO) regarding digital play addiction tendency. Examining these sub-dimensions is important for a more detailed understanding of the origins, triggers, and consequences of aggression. Thus, different intervention and prevention strategies can be developed for each sub-dimension. For example, one child’s aggressive behaviors associated with digital game addiction tendency may require interventions based on regulating digital game use or encouraging alternative social activities. At the same time, different arrangements may be included in the strategies to be implemented for another child. The importance of early intervention for children in the age group we addressed within the scope of the research and the limited number of studies on this subject hinder the strategies to be developed. In this manner, it is also very important to present the results obtained in the research in a theoretical framework to make them more understandable and to provide a foundation suitable for development.

### Current Study

As noted, much of the research on digital gaming and its behavioral correlates has been conducted with older children, particularly preadolescents and adolescents. This focus, while valuable, has created a significant research gap regarding early childhood—a foundational and distinct developmental period. The rationale for focusing on preschoolers is clear: this age involves rapid socio-emotional maturation and the mechanisms of addiction tendency and aggression may manifest differently than in older age groups. Therefore, identifying these relationships at an early age is critical for developing effective intervention strategies.

Building on existing theoretical and empirical work, the present study aims to examine the relationship between digital play addiction tendencies and aggression. Crucially, our investigation focuses on a general digital play addiction tendency—assessing behavioral patterns like loss of control and withdrawal—rather than the effects of specific game content, such as exposure to violent themes. By focusing on the addictive process itself, we seek to understand its links to four distinct sub-dimensions of aggression: physical, relational, self-directed, and aggressive behaviors toward objects. Based on this aim, we generated the following hypotheses:

**H1.**  *Digital play addiction tendency is positively associated with physical aggression.*

**H2.**  *Digital play addiction tendency is positively associated with relational aggression.*

**H3.**  *Digital play addiction tendency is positively associated with self-directed aggression.*

**H4.**  *Digital play addiction tendency is positively associated with aggressive behaviors toward objects.*

**H5.**  *Children’s digital gameplay tendencies predict aggressive behaviors regardless of gender.*

## 2. Materials and Methods

### 2.1. Procedure

This research is a descriptive study aiming to examine the relationship between digital play addiction tendencies and children’s aggression tendencies in early childhood. His study used a quantitative approach, employing a survey and relational design. Cross-sectional data were collected in the spring semester of 2022. Ethics committee approval was obtained from the scientific research ethics committee at Ağrı İbrahim Çeçen University (22 June 2022; Application No: 157). The data for this study were collected online from participants residing in three different cities in Turkey. In each city, the researchers visited state kindergartens and distributed the link to the online survey (hosted on Google Forms) to parents via classroom teachers. Only parents who voluntarily agreed to participate were included in the study sample.

### 2.2. Participants

The study participants consisted of 744 parents, including 610 mothers and 134 fathers, who had children aged 4–6 years. The mothers’ ages ranged from 22 to 55 years (M = 32), while the fathers’ ages ranged from 22 to 58 years (M = 35). Regarding educational background, 22.8% of the mothers had completed primary school, 38% had completed secondary school or high school, 37.1% had attended college or university, and 1.7% held a master’s or doctoral degree. Among fathers, 16% had completed primary school, 37.9% secondary school or high school, 41.8% college or university, and 4.3% held master’s or doctoral degrees. In terms of socioeconomic status, 27% of the families were in the low socioeconomic level group, 37% in the middle, and 36% in the high socioeconomic level group. All the children were attending preschool education at state schools. Data about children’s digital play addiction tendencies and aggressions were obtained through the evaluations of their parents. When examining the demographic information of the study participants, it is notable that 52.4% (n = 390) are male children, while 47.6% (n = 354) are female children. Regarding the ages of the children, 31.6% (n = 235) are 4 years old, 32% (n = 238) are 5 years old, and 36.4% (n = 271) are 6 years old.

### 2.3. Measures

The Aggression Tendency Scale (ATS) was used to evaluate the aggression orientation of 36–72-month-old children. The ATS consists of 27 items divided into four factors: PA, RA, SA, and AO (Kaynak et al., 2016). The ATS includes items such as ‘hits another person (e.g., a friend, sibling, or adult) on purpose to cause harm’ and is rated on a 7-point Likert scale. In this study, Cronbach’s alpha was found to be 0.983.

The Digital Game Addiction Tendency Scale (DPATS) was developed to assess digital game addiction tendencies in early childhood. The DPATS includes items such as ‘gets angry when not allowed to play digital games’ and is rated on a 5-point Likert scale. DPATS consists of 20 items, and in this study, the scale was evaluated as a single factor. The development study of the DPATS confirmed its unidimensional structure, and in the present study, this structure was further verified through confirmatory factor analysis (CFA) (Budak, 2020; Budak & Işıkoğlu, 2022). In this study, Cronbach’s alpha was found to be 0.961.

### 2.4. Data Analysis

SPSS 26 was used for descriptive analysis, and the SmartPLS 4 software was used for structural equation modeling. First, the model was created with PLS path modeling. Then, convergent and discriminant validity were tested to evaluate the measurement model. Finally, the structural model was evaluated by using bootstrapping analysis (5000 resamples). Although the sample size would permit the use of covariance-based SEM, PLS-SEM was chosen because the primary goal of this study was to maximize explained variance and assess predictive relevance rather than to confirm an established theory. Additionally, PLS-SEM is more robust to non-normal data distributions and complex measurement models, making it the most suitable approach for the present research (Hair et al., 2022).

## 3. Results

### 3.1. Preliminary Analysis

Preliminary analyses comprised computing descriptive statistics and correlation coefficients for the analyzed variables. The assumption of a normal distribution was explored using kurtosis and skewness statistics. According to the preliminary analysis results, the data met the necessary assumptions.

### 3.2. Measurement Model Assessment

At the evaluation of the measurement model stage, Cronbach’s alpha, item loadings, CR, and AVE values were checked for convergent validity (see Table 1). Table 1 shows that item loadings showed that the values were in the range of 0.628–0.941 (>0.6). According to Cronbach’s alpha and composite reliability (CR) results, all values were above 0.9. In addition, the AVE values were in the range of 0.577–0.855 (>0.5). Because the model contains only one predictor variable, multicollinearity between predictors was not examined. Therefore, VIF (variance inflation factor) values were examined over the items, and the values met the multicollinearity assumption. After all, considering these values, it is seen that convergent validity was established (Hair et al., 2022).

The discriminant validity stage was assessed using the Fornell-Larcker criterion and the HTMT ratio. Table 2 shows that the results meet the Fornell-Larcker (all square root values of the AVEs of the constructs are greater than the inter-structure correlations) and HTMT ratio (all values are lower than 0.9) criteria (Şahin et al., 2022). Thus, discriminant validity was established (Fornell & Larcker, 1981; Hair et al., 2022). SmartPLS generates only the SRMR (standardized root mean square residual) value from the model fit indices. The SRMR value for the model is 0.045.

### 3.3. Structural Model Assessment

**Figure 1:**
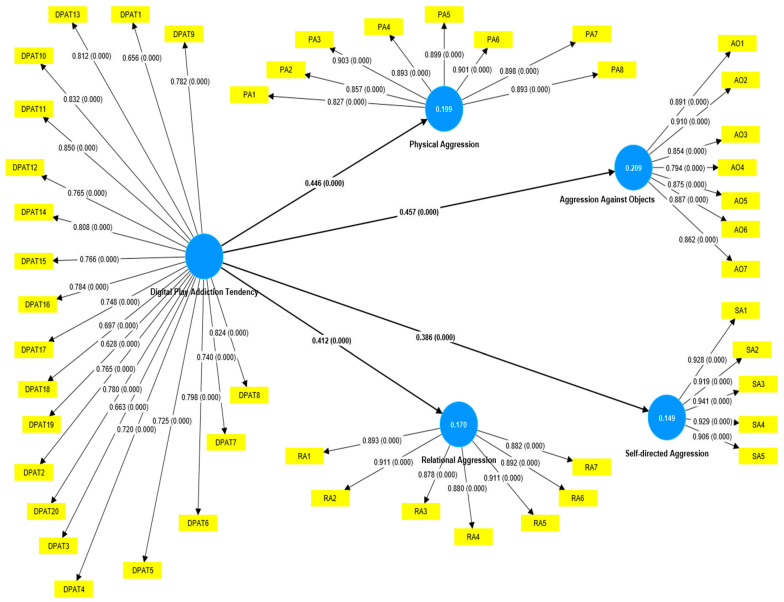
A visual representation of the findings of PLS-SEM.

In this study, children’s aggression tendencies comprised four factors as the dependent variables: PA, RA, SA, and AO. Also, DPAT is a predictor variable (see Table 3). Detailed results of SEM are presented in Figure 1. According to the model, DPAT accounted for 21% (R^2^ = 0.209) of the variance in AO, 20% (R^2^ = 0.199) in PA, 17% (R^2^ = 0.170) in RA, and 15% (R^2^ = 0.149) in SA. These findings suggest that DPAT is a significant predictor of children’s aggression tendencies. It is particularly noteworthy that a single predictor variable was able to explain between 15% and 21% of the variance across different types of aggression. According to structural model results, children’s digital play addiction tendency (DPAT) is a significant predictor of physical aggression toward others (PA) (β = 0.446, *p* < 0.001), relational aggression towards others (RA) (β = 0.412, *p* < 0.001), self-directed aggression (SA) (β = 0.386, *p* < 0.001), and aggression against objects (AO) (β = 0.457, *p* < 0.001). The explanatory power of the model is moderate (R^2^ = 0.149–0.209). The effect sizes (f^2^ = 0.175–0.265) are also moderate, indicating that the DPAT variable has a significant impact on the subdimensions of aggression. The positive Q^2^ values obtained from the blindfolding analysis (0.144–0.204) demonstrate that the model possesses a moderate level of predictive relevance. Overall, the model shows that DPAT significantly and predictively explains all subdimensions of aggression. Results indicated that the increase in children’s digital play addiction tendencies in early childhood causes an increase in children’s aggression tendencies in all types of aggression. To examine whether this result changes according to the gender of the children, a multigroup analysis was performed.

### 3.4. Multigroup Analysis

To perform multi-group analyses, firstly, measurement invariance analyses were performed. In the study, the measurement invariance of composite models (MICOM) method was used to examine the measurement invariance of composite models. This method consists of three steps: configurational invariance, compositional invariance, and equality of composite mean values and variance. Having the same indicators and latent variables within the groups, being the same in terms of reflective structures, and having undergone the same data collection process indicate that configural invariance, the first step of measurement invariance, is achieved. To examine the compositional invariance and equality of composite mean values and variance (scalar invariance), measurement invariance tests were performed (Henseler et al., 2016). The test results are presented in Table 4.

Table 4 indicates no difference between the variables regarding compositional invariance in the measurement invariance tests for gender. Furthermore, no difference was observed for the equality of composite variance for gender. However, some factors related to the equality of composite mean values for gender were not within the appropriate ranges of values. According to these results, configure and compositional measurement invariance were achieved, but scalar measurement invariance could not be achieved. Scalar measurement invariance is required to ensure the validity of mean comparisons across groups. When this level of invariance is not achieved, direct comparisons of group means may yield biased results, as the measurement instruments do not function identically across groups. In our study, full scalar invariance was not established; however, partial measurement invariance was achieved. Partial invariance indicates that, although some items may differ across groups, the overall model remains comparable in its structure. Therefore, structural relationships and parameters can be reliably compared between gender groups (Steinmetz, 2013).

The multi-group analysis found no significant differences between gender groups across all paths in the model. This indicates that digital play addiction tendency in early childhood negatively impacts the social behavior development of all children, regardless of gender.

## 4. Discussion

This study aimed to investigate the relationship between digital play addiction tendencies and aggression among children in early childhood. Our hypothesis that digital play addiction tendency would correlate positively with various dimensions of aggression (PA, RA, SA, AO) was supported by the findings. The results indicate that increased digital play addiction tendencies in early childhood are associated with higher levels of aggression across all sub-dimensions (PA, RA, SA, AO). The stress, competition, and reward systems inherent in digital games may lead children to employ less effective real-world problem-solving strategies, potentially translating aggressive behaviors experienced in digital contexts into real-life scenarios. Therefore, it can be argued that digital play addiction tendency is associated with physical aggression (PA).

Furthermore, our findings reveal an association between digital play addiction tendency and self-directed aggression (SA), where emotional responses to setbacks or challenges in digital games manifest as self-directed hostility rather than healthy coping mechanisms. In terms of relational aggression (RA), digital play addiction tendency may be associated with challenges in children’s social skills development, affecting their ability to form positive relationships with peers and family members. Competitive environments within digital games may teach aggressive competition and conflict resolution strategies that spill over into real-world relational dynamics. Additionally, aggression against objects (AO) appears to be associated with digital play addiction tendency, as success or failure within games may evoke emotional responses such as anger, which are reflected in aggressive behaviors toward objects in the physical environment.

Our findings are consistent with prior research, reinforcing the link between digital play addiction tendency and detrimental outcomes, particularly aggressive behaviors in children (Gentile et al., 2011). Previous studies have consistently demonstrated a heightened tendency for aggression among children addicted to digital play (Jeong et al., 2017; Lee & Morgan, 2018). Contributing factors include excessive and unsupervised digital gameplay, which is associated with impairments in children’s emotional regulation and behavioral control, and related to aggression (Schettler et al., 2024). Furthermore, children addicted to digital play may experience heightened emotional arousal and aggression when access to digital devices is restricted (Seo & Lim, 2010; Şenol et al., 2023). These findings underscore the impact of impulsivity and emotional regulation deficits in exacerbating aggressive behaviors following limitations on digital play.

Another critical aspect highlighted in the socio-emotional context is the development of executive functions, particularly skills related to emotion regulation. While social games facilitate core executive functions such as emotion regulation, inhibitory control (self-control), and behavior regulation through interactions with peers, these skills may be less cultivated in digital gaming environments (Işıkoğlu et al., 2023). The expression of negative emotions during digital gameplay appears to correspond with a higher incidence of conflict between children and their parents. A possible explanation is that digital environments, often lacking the nuanced feedback of peer interaction necessary for developing cognitive flexibility, may limit opportunities for children to practice emotional regulation. Therefore, there is a possibility that challenges in executive functioning, cultivated during gameplay, could manifest beyond the digital context, shaping social interactions and behavioral patterns in the real world.

Moreover, the reciprocal relationship between aggression and digital play addiction tendency suggests a mutual reinforcement. Research indicates that aggressive behaviors may enhance engagement in digital games, where achieving goals and rewards intensifies goal-oriented aggression (Ko et al., 2009; Mehroof & Griffiths, 2010). The activation of aggressive content in digital games can also be associated with neurological responses and potentially related to real-life aggressive tendencies among children (Weber et al., 2006).

This study reveals that, regardless of gender, children addicted to digital play exhibit higher levels of aggression. Our findings align with previous research (Hastings et al., 2009), indicating that gender does not significantly mediate addiction trends in digital gaming. However, further investigation into gender-specific factors related to digital play addiction tendency and its association with social skills may provide deeper insights (Ekinci et al., 2016; Hazar, 2019).

### 4.1. Study Limitations

Existing literature regarding participatory child research has criticized studies that examine variables related to children using only adult evaluations, without including the children’s own perspectives. In this study, digital play addiction tendencies and aggressive behaviors of children in early childhood were examined with their parents’ evaluations. Examining the definition of digital play addiction tendency, it is necessary to observe behaviors such as digital games taking precedence over the child’s behaviors, the frequency of playing digital games, digital games becoming a primary means of entertainment, conflicts with the environment, and lying to continue playing digital games. In large-scale quantitative studies such as this study, researchers cannot observe such situations. Considering that children do not use digital games in the school environment, the most appropriate people to observe and evaluate children’s use of digital technology at home are their parents. Thus, although relying on parental reports is a limitation, the researchers preferred this method to ensure more reliable data, as parents are best positioned to observe these home-based behaviors.

Despite the justifiable reliance on parental reports, two significant methodological limitations must be acknowledged. First, the exclusive reliance on a single source of data collection introduces the risk of common method bias, which may artificially inflate the observed associations between the measured constructs (digital play tendency and aggression). Although measures were selected and administered with care, future studies should endeavor to incorporate multiple informants (e.g., teachers, peers) to mitigate this potential bias. Second, due to the cross-sectional design of this research, definitive conclusions regarding causality are precluded. While our Structural Equation Model suggests a predictive path, the findings should not be interpreted as establishing a causal effect. We must acknowledge the possibility of reverse causation, where high levels of existing aggressive behavior could be associated with increased engagement with and dependency on digital play. Longitudinal studies are therefore essential to determine the temporal precedence of this relationship.

### 4.2. Implications for Practice

Changes in digital technology have made digital games an integral part of our lives. This makes digital play addiction tendency, which is also associated with psychosocial factors such as loneliness, depression, and aggression, one of today’s serious health problems (Jeong et al., 2017). Considering that it is not possible to keep children away from digital play today, it is thought that the best strategy to minimize the harm is to guide children by their parents (Şenol et al., 2023). Digital technology and its benefits of this technology have an important place in children’s lives today. Considering this, it is inevitable to conclude that digital technology and digital play are effective in shaping children’s development and later years. With interventions from an early age, children can become more conscious digital technology users in the future. To protect children from digital play addiction tendency and violent content, digital games played by young children must be under parental supervision, and game developers can consider scientific studies in the relevant field. Age restrictions can be imposed on games that may have negative effects on young children. In addition, families and educators have a great responsibility to ensure that violence and aggression are not normalized and that resorting to violence as a way of solving problems in daily life is not perceived as normal behavior.

Finally, it is important to interpret these findings with caution, as the cross-sectional nature of the study does not allow for firm conclusions about causality. The possibility of reverse causation should also be acknowledged—that is, rather than digital play addiction tendency being associated with aggression, children who already show higher levels of aggression might also be more likely to engage in digital play. Future research employing longitudinal or experimental designs will be valuable for clarifying the direction of this relationship and informing more targeted preventive interventions.

## 5. Conclusions

In conclusion, this study contributes to understanding the relationships between digital play addiction tendency and aggression among children in early childhood. It highlights the need for parental awareness and early interventions to mitigate the adverse effects of digital gaming, emphasizing the importance of promoting healthy digital habits and socio-emotional development from a young age.

## Figures and Tables

**Table 1:** Convergent Validity.

Constructs	Item	Loading	VIF	α	CR	AVE
AO	AO1	0.891	4.103	0.945	0.955	0.754
	AO2	0.910	4.624			
	AO3	0.854	2.937			
	AO4	0.794	2.260			
	AO5	0.875	3.883			
	AO6	0.887	4.147			
	AO7	0.862	3.900			
PA	PA1	0.827	2.665	0.960	0.966	0.782
	PA2	0.857	2.941			
	PA3	0.903	4.068			
	PA4	0.893	4.331			
	PA5	0.899	4.441			
	PA6	0.901	4.419			
	PA7	0.898	4.255			
	PA8	0.893	4.070			
RA	RA1	0.893	3.804	0.957	0.965	0.797
	RA2	0.911	4.588			
	RA3	0.878	3.363			
	RA4	0.880	3.375			
	RA5	0.911	4.388			
	RA6	0.892	3.777			
	RA7	0.882	3.590			
SA	SA1	0.928	5.182	0.958	0.967	0.855
	SA2	0.919	4.293			
	SA3	0.941	5.967			
	SA4	0.929	4.770			
	SA5	0.906	3.619			
DPAT	DPAT1	0.656	1.893	0.961	0.964	0.577
	DPAT10	0.832	3.545			
	DPAT11	0.850	4.394			
	DPAT12	0.765	2.394			
	DPAT13	0.812	3.004			
	DPAT14	0.808	3.386			
	DPAT15	0.766	2.854			
	DPAT16	0.784	2.998			
	DPAT17	0.748	2.425			
	DPAT18	0.697	2.347			
	DPAT19	0.628	1.925			
	DPAT2	0.765	2.746			
	DPAT20	0.780	2.885			
	DPAT3	0.663	2.052			
	DPAT4	0.720	2.279			
	DPAT5	0.725	2.406			
	DPAT6	0.798	2.733			
	DPAT7	0.740	2.810			
	DPAT8	0.824	4.099			
	DPAT9	0.782	3.097			

**Table 2:** Fornell-Larcker Criterion (discriminant validity) and HTMT Ratio (discriminant validity).

Fornell-Larcker Criterion					
Variable	AO	DPAT	RA	PA	SA
AO	0.868				
DPAT	0.457	0.759			
RA	0.820	0.412	0.893		
PA	0.845	0.446	0.863	0.884	
SA	0.867	0.386	0.859	0.832	0.925
HTMT ratio					
	AO	DPAT	PA	RA	SA
AO					
DPAT	0.470				
PA	0.888	0.455			
RA	0.865	0.420	0.895		
SA	0.898	0.392	0.868	0.897	

**Table 3:** Structural modelling results.

Path	Coefficient	t Value	R^2^	Adjusted R^2^	f^2^	Q^2^
DPAT -> AO	0.457	15.431 ***	0.209	0.208	0.265	0.204
DPAT -> PA	0.446	13.886 ***	0.199	0.198	0.248	0.194
DPAT -> RA	0.412	13.382 ***	0.170	0.169	0.205	0.165
DPAT -> SA	0.386	12.426 ***	0.149	0.148	0.175	0.144

*** *p* < 0.01.

**Table 4:** Measurement invariance results.

**Compositional Invariance for Gender**					
Factor	Original correlation	Correlation permutation mean		5.0%	Permutation *p*-value
AO	1.000	1.000		0.999	0.260
DPAT	1.000	1.000		0.999	0.798
IS	1.000	1.000		1.000	0.650
PA	1.000	1.000		1.000	0.906
SA	1.000	1.000		1.000	0.800
**Equality of Composite Mean Values for Gender**					
Factor	Original difference	Permutation mean difference	2.5%	97.5%	Permutation *p*-value
AO	−0.122	0.003	−0.133	0.141	0.085
DPAT	−0.170	0.000	−0.137	0.141	0.016
IS	−0.022	0.005	−0.134	0.150	0.751
PA	−0.110	0.003	−0.134	0.146	0.116
SA	−0.059	0.004	−0.136	0.149	0.393
**Equality of Composite Variance for Gender**					
Factor	Original difference	Permutation mean difference	2.5%	97.5%	Permutation *p*-value
AO	−0.030	0.006	−0.220	0.254	0.816
DPAT	−0.033	−0.004	−0.160	0.150	0.672
IS	−0.033	0.010	−0.223	0.257	0.803
PA	0.009	0.007	−0.226	0.250	0.940
SA	−0.012	0.009	−0.245	0.291	0.925

## Data Availability

The original contributions presented in this study are included in the article. Further inquiries can be directed to the corresponding authors.

## References

[B1-ejihpe-15-00233] American Academy of Pediatrics [AAP] Council of Communications and Media (2016). Media and young minds. Pediatrics.

[B2-ejihpe-15-00233] Bandura A. (1986). Social foundations of thought and action *(p. 2)*.

[B3-ejihpe-15-00233] Bandura A. (2001). Social cognitive theory: An agentic perspective. Annual Review of Psychology.

[B4-ejihpe-15-00233] Boot W. R., Kramer A. F., Simons D. J., Fabiani M., Gratton G. (2008). The effects of video game playing on attention, memory, and executive function. Acta Psychologica.

[B5-ejihpe-15-00233] Budak K. S. (2020). Okul öncesi dönem çocukları için dijital oyun bağımlılık eğilimi ölçeğinin ve dijital oyun ebeveyn rehberlik stratejileri ölçeğinin geliştirilmesi, problem davranışlarla ilişkisinin incelenmesi. [Unpublished master’s thesis].

[B6-ejihpe-15-00233] Budak K. S., Işıkoğlu N. (2022). Development of children’s digital play addiction tendency and parental mediation scales. Ankara University Journal of Faculty of Educational Sciences (JFES).

[B7-ejihpe-15-00233] Caner N., Evgin D. (2021). Digital risks and adolescents: The relationships between digital game addiction, emotional eating, and aggression. International Journal of Mental Health Nursing.

[B8-ejihpe-15-00233] Cowan K., Potter J., Olusoga Y., Bannister C., Bishop J. C., Cannon M., Signorelli V. (2021). Children’s digital play during the COVID-19 pandemic: Insights from the play observatory. Je-LKS: Journal of e-Learning and Knowledge Society.

[B9-ejihpe-15-00233] Ekinci N. E., Ustun U. D., Ozer O. (2016). An investigation of the relationship between digital game addiction, gender, and regular sports participation. Journal of Educational and Cultural Studies.

[B10-ejihpe-15-00233] Emiroğlu İlvan T., Ceylan R. (2023). Predicting preschool children’s digital play addiction tendency during Covid-19 pandemic: Regarding the mother-child relationship, and child- and family-related factors. Education and Information Technologies.

[B11-ejihpe-15-00233] Fornell C., Larcker D. F. (1981). Evaluating structural equation models with unobservable variables and measurement error. Journal of Marketing Research.

[B12-ejihpe-15-00233] Gansner M. E. (2019). Gaming addiction in ICD-11: Issues and implications. Psychiatric Times.

[B13-ejihpe-15-00233] Gentile D. A., Choo H., Liau A., Sim T., Li D., Fung D., Khoo A. (2011). Pathological video game use among youths: A two-year longitudinal study. Pediatrics.

[B14-ejihpe-15-00233] Granic I., Morita H., Scholten H. (2020). Beyond screen time: Identity development in the digital age. Psychological Inquiry.

[B15-ejihpe-15-00233] Hair J. F., Hult G. T. M., Ringle C. M., Sarstedt M. (2022). A primer on partial least squares structural equation modeling (PLS-SEM).

[B16-ejihpe-15-00233] Hastings E. C., Karas T. L., Winsler A., Way E., Madigan A., Tyler S. (2009). Young children’s video/computer game use: Relations with school performance and behavior. Issues in Mental Health Nursing.

[B17-ejihpe-15-00233] Hazar Z. (2019). An analysis of the relationship between digital game playing motivation and digital game addiction among children. Asian Journal of Education and Training.

[B18-ejihpe-15-00233] Henseler J., Ringle C. M., Sarstedt M. (2016). Testing measurement invariance of composites using partial least squares. International Marketing Review.

[B19-ejihpe-15-00233] Hirsh-Pasek K., Golinkoff R. M., Berk L. E., Singer D. G. (2009). A mandate for playful learning in preschool: Presenting the evidence.

[B20-ejihpe-15-00233] Işıkoğlu N., Erol A., Atan A., Aytekin S. (2023). A qualitative case study about the overuse of digital play at home. Current Psychology.

[B21-ejihpe-15-00233] Jeong E. J., Kim D. J., Lee D. M. (2017). Why do some people become addicted to digital games more easily? A study of digital game addiction from a psychosocial health perspective. International Journal of Human–Computer Interaction.

[B22-ejihpe-15-00233] Johnston K. (2021). Engagement and immersion in digital play: Supporting young children’s digital wellbeing. International Journal of Environmental Research and Public Health.

[B23-ejihpe-15-00233] Kaynak B., Kan A., Kurtulmuş Z. (2016). Development of “aggression tendency scale for 36–72 months-old children”. Journal of Turkish Studies.

[B24-ejihpe-15-00233] Kesici A. (2020). The effect of conscientiousness and gender on digital game addiction in high school students. Journal of Education and Future.

[B25-ejihpe-15-00233] Ko C. H., Yen J. Y., Liu S. C., Huang C. F., Yen C. F. (2009). The associations between aggressive behaviors and internet addiction and online activities in adolescents. Journal of Adolescent Health.

[B26-ejihpe-15-00233] Koran N., Berkmen B., Adalıer A. (2022). Mobile technology usage in early childhood: Pre-COVID-19 and the national lockdown period in North Cyprus. Education and Information Technologies.

[B27-ejihpe-15-00233] Lee G. L., Morgan H. (2018). Understanding children’s attraction toward digital games and preventing their gaming addiction. US-China Education Review A.

[B28-ejihpe-15-00233] Limone P., Toto G. (2021). Psychological and emotional effects of digital technology on children in COVID-19 pandemic. Brain Sciences.

[B29-ejihpe-15-00233] Marsh J., Murris K., Ng’ambi D., Parry R., Scott F., Thomsen B. S., Bishop J., Bannister C., Dixon K., Giorza T., Peers J., Titus S., Da Silva H., Doyle G., Driscoll A., Hall L., Hetherington A., Krönke M., Margary T., Woodgate A. (2020). Children, technology and play..

[B30-ejihpe-15-00233] Mehroof M., Griffiths M. D. (2010). Online gaming addiction: The role of sensation seeking, self-control, neuroticism, aggression, state anxiety, and trait anxiety. Cyberpsychology, Behavior, and Social Networking.

[B31-ejihpe-15-00233] Mhurchú C. N., Maddison R., Jiang Y., Jull A., Prapavessis H., Rodgers A. (2008). Couch potatoes to jumping beans: A pilot study of the effect of active video games on physical activity in children. International Journal of Behavioral Nutrition and Physical Activity.

[B32-ejihpe-15-00233] Nagar K., Patidar A. K., Mudgal S. K., Gaur R., Patidar V. (2025). Relationship of digital game addiction with aggression and anger in the post COVID-19 era: A systematic review and meta-analysis. Investigación y Educación en Enfermería.

[B33-ejihpe-15-00233] Organisation for Economic Co-Operation and Development (OECD) (2019). What do we know about children and technology?.

[B34-ejihpe-15-00233] Palaiologou I. (2016). Children under five and digital technologies: Implications for early years pedagogy. European Early Childhood Education Research Journal.

[B35-ejihpe-15-00233] Prescott A. T., Sargent J. D., Hull J. G. (2018). Meta-analysis of the relationship between violent video game play and physical aggression over time. Proceedings of the National Academy of Sciences of the United States of America.

[B36-ejihpe-15-00233] Rahayu I. S., Karana I., Hardiansyah M. A., Dewi D. H., Elihami E. (2021). The relationship of online game addiction with learning motivation in school age children on COVID-19 pandemic. Linguistics and Culture Review.

[B37-ejihpe-15-00233] Schettler L. M., Thomasius R., Paschke K. (2024). Emotional dysregulation predicts problematic gaming in children and youths: A cross-sectional and longitudinal approach. European Child & Adolescent Psychiatry.

[B38-ejihpe-15-00233] Seo M. Y., Lim E. M. (2010). Infants’ and low-grade elementary students’ internet game addiction tendency and the relationship between game addiction tendency and personality characteristic. The Journal of Child Education.

[B39-ejihpe-15-00233] Steinmetz H. (2013). Analyzing observed composite differences across groups: Establishing partial measurement invariance by means of partial scalar invariance. Methods, Data, Analyses.

[B40-ejihpe-15-00233] Şahin F., Doğan E., Yıldız G., Okur M. R. (2022). University students with special needs: Investigating factors influencing e-learning adoption. Australasian Journal of Educational Technology.

[B41-ejihpe-15-00233] Şenol Y., Şenol F. B., Can Yaşar M. (2023). Digital game addiction of preschool children in the COVID-19 pandemic: Social emotional development and parental guidance. Current Psychology.

[B42-ejihpe-15-00233] Taylan H., Kara H., Durğun A. (2017). Ortaokul ve lise öğrencilerinin bilgisayar oyunu oynama alışkanlıkları ve oyun tercihleri üzerine bir araştırma. PESA Uluslararası Sosyal Araştırmalar Dergisi.

[B43-ejihpe-15-00233] Tena R. R., Gutiérrez M. P., Cejudo M. D. C. L. (2019). Technology use habits of children under six years of age at home. Ensaio: Avaliação e Políticas Públicas em Educação.

[B44-ejihpe-15-00233] Turgut M., Yaşar O. M. (2019). Playing digital game motivations of university students. Asian Journal of Education and Training.

[B45-ejihpe-15-00233] Turkish Statistical Institute (2013). Hanehalkı bilişim teknolojileri (BT) kullanım araştırması.

[B46-ejihpe-15-00233] Turkish Statistical Institute (2021a). Çocuklarda bilişim teknolojileri kullanım araştırması.

[B47-ejihpe-15-00233] UNICEF (2025). Calling for play in an increasingly digital world.

[B48-ejihpe-15-00233] Weber R., Ritterfeld U., Mathiak K. (2006). Does playing violent video games induce aggression? Empirical evidence of a functional magnetic resonance imaging study. Media Psychology.

[B49-ejihpe-15-00233] Weinstein A. M. (2010). Computer and video game addiction: A comparison between game users and non-game users. The American Journal of Drug and Alcohol Abuse.

[B50-ejihpe-15-00233] Žulec A., Merkaš M., Varga V. (2023). Screen-based activities among children in Croatia: A media diary approach. Journal of Psychological & Educational Research.

